# Supplementary material for: SURGE: uncovering context-specific genetic-regulation of gene expression from single-cell RNA sequencing using latent-factor models
Source: Genome Biol. 2024 Jan 22;25:28. doi: 10.1186/s13059-023-03152-z (PMC10801966; doi:10.1186/s13059-023-03152-z)
Supplement: Supplementary file 1 — Additional file 1: Supplementary methods. Fig. S1. Computational runtime of SURGE latent context inference and SURGE interaction eQTL calling. Fig. S2. Evaluation of SURGE’s ability to re-capture simulated latent contexts simulations. Fig. S3. Evaluation of SURGE’s ability to identify correct number of simulated latent contexts in simulations. Fig. S4. Proportion of expression variance explained by SURGE latent contexts when SURGE was applied to samples concatenated across 10 GTEx v8 tissues. Fig. S5. Q-Q plot for SURGE interaction eQTLs identified when SURGE was applied to samples concatenated across 10 GTEx v8 tissues. Fig. S6. Q-Q plot for SURGE interaction eQTLs relative to expression PC interaction eQTLs when SURGE was applied to samples concatenated across 10 GTEx v8 tissues. Fig. S7. Comparison of inferred SURGE latent contexts with and without random effects included when SURGE was applied to samples concatenated across 10 GTEx v8 tissues. Fig. S8. SURGE latent context 4 and SURGE latent context 7 are explained by genotype PC1 when SURGE was applied to samples concatenated across 10 GTEx v8 tissues. Fig. S9. Relationship between SURGE latent contexts and xCell cell type enrichment score when SURGE was applied to samples concatenated across 10 GTEx v8 tissues. Fig. S10. Relationship between SURGE latent context 5 and Epithelial cell type enrichment score, and SURGE latent context 6 and Neuron cell type enrichment score. Fig. S11. Correlation between SURGE latent contexts and gene expression principal components and genotype principal components when SURGE was applied to samples concatenated across 10 GTEx v8 tissues. Fig. S12. Relationship between SURGE latent context 1 and xCell cell type enrichment score when SURGE was applied to samples from Colon-Sigmoid GTEx v8 tissue. Fig. S13. P values of SURGE latent context 1 interaction eQTLs when SURGE was applied to samples from only Colon-Sigmoid GTEx v8 tissue compared to pvalues of interaction eQTLs using xCell [file 13059_2023_3152_MOESM1_ESM.docx]

Additional file 1 for:

SURGE: uncovering context-specific genetic-regulation of gene expression from single-cell RNA-sequencing using latent-factor models

Benjamin J. Strober^1^, Karl Tayeb^2^, Joshua Popp^3^, Guanghao Qi^3^, M. Grace Gordon^4,5,6,7^, Richard Perez^6^, Chun Jimmie Ye^5,6,7,8,9^, Alexis Battle^3,10,11,&^

1. Department of Epidemiology, Harvard T.H. Chan School of Public Health, Boston, MA, USA

2. Department of Human Genetics, University of Chicago, Chicago, Illinois, USA

3. Department of Biomedical Engineering, Johns Hopkins University, Baltimore, MD,

4. Biological and Medical Informatics Graduate Program, University of California, San Francisco, CA, USA

5. Division of Rheumatology, Department of Medicine, University of California, San Francisco, CA, USA

6. Institute for Human Genetics, University of California, San Francisco, CA, USA

7. Department of Bioengineering and Therapeutic Sciences, University of California, San Francisco, CA, USA

8. Institute for Computational Health Sciences, University of California, San Francisco, San Francisco, CA, USA

9. Chan-Zuckerberg Biohub, CA, USA

10. Department of Computer Science, Johns Hopkins University, Baltimore, Maryland, USA

11. Department of Genetic Medicine, Johns Hopkins University, Baltimore, Maryland, USA

& Corresponding Author: ajbattle@jhu.edu

**SURGE model overview**

The SURGE model is defined according to the following probability distributions:

$$y_{nt}\sim N(\mu_{t}+\sum_{l} X_{nl}W_{lt}+\sum_{i} I\left[ n\in i \right]\alpha_{it}+G_{nt}F_{t}+G_{nt}(\sum_{k} U_{nk}V_{kt}), \sigma_{t}^{2})$$

$$U_{nk}\sim N\left( 0, \gamma_{k}^{2} \right)$$

$$V_{kt}\sim N\left( 0,1 \right)$$

$$1/\gamma_{k}^{2}\sim Gamma(\alpha_{0},\beta_{0})$$

$$F_{t}\sim N\left( 0,1 \right)$$

$$\alpha_{it}\sim N(0,\psi_{t}^{2})$$

$${1/\psi}_{t}^{2}\sim Gamma\left( \alpha_{0}, \beta_{0} \right)$$

$${1/\sigma}_{t}^{2}\sim Gamma(\alpha_{0}, \beta_{0})$$

Here, $n$ indexes RNA samples, $t$ indexes representative variant-gene pairs being tested for eQTL analysis, and $i$ indexes individuals. We use the notation $n\in i$ to represent the instance where RNA sample $n$ is drawn from the individual $i$. $y_{nt}$ is the observed normalized gene expression (mean 0 and variance 1 for each test $t$) level of the gene corresponding to test $t$ in sample $n$. $G_{nt}$ is the observed, standardized genotype of the variant corresponding to test $t$ in sample $n$. $X_{nl}$ is the observed value of covariate $l$ for sample $n$ .

To standardize the genotype of the variant corresponding to test $t$, we center the genotype vector to have mean 0 across samples and then we scale the genotype vector for test $t$ ($G_{*t}$) by the standard deviation of $Y_{*t}/G_{*t}$. This scaling encourages the low-dimensional factorization ($UV$) to explain variance equally across tests instead of preferentially explaining variance in tests with small variance in $Y_{*t}/G_{*t}$.

SURGE infers the values of:

- $F_{t}$: the eQTL effect size of test $t$ that is shared across samples
- $V_{kt}$: the eQTL effect size of test $t$ for latent context $k$
- $U_{nk}$: the latent context value of sample $n$ on factor $k$
- $\mu_{t}$: the intercept of each test
- $W_{lt}$: The effect size of covariate $l$ on the gene corresponding to test $t$
- $\alpha_{it}$: the random effect intercept for each individual for each test
- $\gamma_{k}^{2}$: The variance of the values in latent context $k$
- $\psi_{t}^{2}$: The variance of intercept corresponding to each individual in test $t$
- $\sigma_{t}^{2}$: The residual variance in gene expression levels in test $t$

$a_{0}$, and $\beta_{0}$ are model hyper-parameters set to provide non-informative priors while stabilizing optimization. In practice we set $\alpha_{0}$ to 1e^-3^ and $\beta_{0}$ to 1e^-3^.

**SURGE inference overview**

We approximate the posterior distribution of all latent variables [$Z=$($F_{t}$, $V_{kt}$, $U_{nk}$, $\mu_{t}$, $W_{lt}$, $\alpha_{it}$, $\gamma_{k}^{2}$, $\psi_{t}^{2}$, $\sigma_{t}^{2}$)] using mean-field variational inference. Variational inference seeks to minimize the KL-divergence from an approximating distribution $q(Z)$ to the exact posterior $p(Z|Y,G,X)$. We used the “mean-field approximation” for $q(Z)$ such that all latent variables are independent of one another. More specifically:

$$\log q\left( Z \right)=$$

$$\sum_{t} \sum_{k} logN\left( V_{kt} \right|\mu_{V_{kt}}, \sigma_{V_{kt}}^{2})+$$

$$\sum_{t} \sum_{i} logN(\alpha_{it}\left| \mu_{\alpha_{it}},\sigma_{\alpha_{it}}^{2} \right)+$$

$$\sum_{t} \sum_{l} \log N\left( W_{lt} | \mu_{W_{lt}}, \sigma_{W_{lt}}^{2} \right)+$$

$$\sum_{t} [ logN\left( F_{t} | \mu_{F_{t}}, \sigma_{F_{t}}^{2} \right)+logN\left( \mu_{t} | \mu_{\mu_{t}}, \sigma_{\mu_{t}}^{2} \right)+logG\left( 1/\psi_{t}^{2} | \alpha_{\psi_{t}},\beta_{\psi_{t}} \right)+logG\left( 1/\sigma_{t}^{2} | \alpha_{\sigma_{t}}, \beta_{\sigma_{t}} \right)]+$$

$$\sum_{k} logG({1/\gamma}_{k}^{2}|\alpha_{\gamma_{k}}, \beta_{\gamma_{k}})+$$

$$\sum_{n} \sum_{k} log N\left( U_{nk} \right|\mu_{U_{nk}}, \sigma_{U_{nk}}^{2})$$

Where $N\left( x \right|\mu, \sigma^{2})$ is a univariate normal distribution parameterized by mean $\mu$ and variance $\sigma^{2}$ and $G(X|\alpha, \beta)$ is a univariate gamma distribution parameterized by $\alpha$ and $\beta$.

It can be shown that minimizing the KL-divergence $KL(q(Z)||p(Z|Y,G,X)$ is equivalent to maximizing the evidence lower bound (ELBO):

$$E_{q}\left[ logp\left( G, Y, X, Z \right) \right]-E_{q}[logq\left( Z \right)]$$

The approach we take to maximize the ELBO is through coordinate ascent, iteratively updating the variational distribution each latent variable, while holding the variational distributions of all other latent variables fixed. Accordingly, the ELBO is guaranteed to monotonically increase after each variational update. In the case of the SURGE model, each update is available in closed form (shown below).

**SURGE coordinate ascent variational inference update equations**

Below we give the closed form update for each latent variable in SURGE, which is applied at each iteration of the coordinate ascent variational inference algorithm. We use the notation $\langle z\rangle$ to represent the expected value of the random value z with respect to the variational distribution ($\left\langle z \right\rangle=E_{q}[z]$)

Latent contexts ($U_{nk})$

For each sample $n$ and latent context $k$,

Prior distribution: $p\left( U_{nk} \right)\sim N(0,\gamma_{k}^{2})$

Variational distribution: $q\left( U_{nk} \right)\sim N({\mu_{U}}_{nk},{\sigma_{U}^{2}}_{nk})$

where the updates are:

$${\sigma_{U}^{2}}_{nk}=\left( ( \right.\sum_{t} \frac{1}{\left\langle\sigma_{t}^{2}\rangle\right.}G_{nt}^{2}{\langle V}_{kt}^{2}\rangle)+ \left. \frac{1.0}{\gamma_{k}^{2}} \right)^{-1}$$

$${\mu_{U}}_{nk}={\sigma_{U}^{2}}_{nk}\sum_{t} \frac{1.0}{\langle\sigma_{t}^{2}\rangle}G_{nt}\langle V_{kt}\rangle\left( r_{nt}^{U_{nk}} \right)$$

$$r_{nt}^{U_{nk}}=Y_{nt}-{\langle\mu}_{t}\rangle-\sum_{l} X_{nl}\left\langle W_{lt} \right\rangle-\sum_{i} I\left[ n\in i \right]{\langle\alpha}_{it}\rangle-G_{nt}{\langle F}_{t}\rangle-G\_nt \sum_{j!=k} \langle U_{nj}\rangle\langle V_{jt}\rangle$$

Latent contexts eQTL effect sizes ($V_{kt})$

For each test $t$ and latent context $k$

Prior distribution: $p\left( V_{kt} \right)\sim N(0, 1)$

Variational distribution $q\left( V_{kt} \right)\sim N({\mu_{V}}_{kt},\sigma_{V_{kt}}^{2})$

where the updates are:

$${\sigma_{V}^{2}}_{kt}=\left( \frac{1.0}{{\langle\sigma}_{t}^{2}\rangle}\sum_{n} G_{nt}^{2}\left\langle U_{nk}^{2} \right\rangle+1 \right)^{-1}$$

$${\mu_{V}}_{kt}={\sigma_{V}^{2}}_{kt}\sum_{n} \frac{1.0}{\langle\sigma_{t}^{2}\rangle}G_{nt}\langle U_{nk}\rangle\left( r_{nt}^{V_{kt}} \right)$$

$$r_{nt}^{V_{kt}}=Y_{nt}-{\langle\mu}_{t}\rangle-\sum_{l} X_{nl}\left\langle W_{lt} \right\rangle-\sum_{i} I\left[ n\in i \right]{\langle\alpha}_{it}\rangle-G_{nt}{\langle F}_{t}\rangle- G_{nt}\sum_{j!=k} \langle U_{nj}\rangle\langle V_{jt}\rangle$$

Shared eQTL effect sizes ($F_{t})$

For each test *t*

Prior distribution: $p\left( F_{t} \right)\sim N(0, 1)$

Variational distribution $q\left( F_{t} \right)\sim N({\mu_{F}}_{t},\sigma_{F_{t}}^{2})$

Where updates are:

$${\sigma_{F}^{2}}_{t}=\left( \frac{1.0}{\langle\sigma_{t}^{2}\rangle}\sum_{n} G_{nt}^{2}+1 \right)^{-1}$$

$${\mu_{F}}_{t}=\sigma_{F_{t}}^{2}\sum_{n} \frac{1}{\left\langle\sigma_{t}^{2} \right\rangle}G_{nt}(r_{nt}^{F_{t}})$$

$$r_{nt}^{F_{t}}=Y_{nt}-{\langle\mu}_{t}\rangle-\sum_{l} X_{nl}\left\langle W_{lt} \right\rangle-\sum_{i} I\left[ n\in i \right]{\langle\alpha}_{it}\rangle- G_{nt}\sum_{k} \langle U_{nk}\rangle\langle V_{kt}\rangle$$

Effects of known covariates on gene expression ($W_{lt})$

For each covariate *l* and each test *t*

Prior distribution: $p\left( W_{lt} \right)\sim N\left( 0, M \right)$

Note M is picked to be infinitely large such that $\frac{1}{M}=0$. This provides no regularization on the effects of covariates and allows as much expression variation as possible to be captured by covariates, leading to conservative estimates of genetic effects on gene expression.

Variational distribution $q\left( W_{lt} \right)\sim N({\mu_{W}}_{lt},{\sigma_{W}^{2}}_{lt})$

Where updates are:

$${\sigma_{W}^{2}}_{lt}=\left( \frac{1.0}{\langle\sigma_{t}^{2}\rangle}\sum_{n} X_{nl}^{2} \right)^{-1}$$

$${\mu_{W}}_{lt}=\sigma_{W_{lt}}^{2}\sum_{n} \frac{1}{\left\langle\sigma_{t}^{2} \right\rangle}X_{nl}(r_{nt}^{W_{lt}})$$

$$r_{nt}^{W_{lt}}=Y_{nt}-{\langle\mu}_{t}\rangle-\sum_{j!= l} X_{nj}\left\langle W_{jt} \right\rangle-\sum_{i} I\left[ n\in i \right]{\langle\alpha}_{it}\rangle-G_{nt}{\langle F}_{t}\rangle- G_{nt}\sum_{k} \langle U_{nk}\rangle\langle V_{kt}\rangle$$

Expression intercept ($\mu_{t})$

For each test *t*

Prior distribution: $p\left( \mu_{t} \right)\sim N\left( 0, M \right)$

Note M is picked to be infinitely large such that $\frac{1}{M}=0$. This provides no regularization on the intercept and allows as much expression variation as possible to be captured by the intercept, leading to conservative estimates of genetic effects on gene expression.

Variational distribution $q\left( \mu_{t} \right)\sim N({\mu_{\mu}}_{t},{\sigma_{\mu}^{2}}_{t})$

Where updates are:

$${\sigma_{\mu}^{2}}_{t}=\left( \frac{N}{\langle\sigma_{t}^{2}\rangle} \right)^{-1}$$

$$\mu_{\mu_{t}}={\sigma_{\mu}^{2}}_{t}\sum_{n} \frac{1}{\left\langle\sigma_{t}^{2} \right\rangle}(r_{nt}^{\mu_{t}})$$

$$r_{nt}^{\mu_{t}}=Y_{nt}-\sum_{l} X_{nl}\left\langle W_{lt} \right\rangle-\sum_{i} I\left[ n\in i \right]{\langle\alpha}_{it}\rangle-G_{nt}{\langle F}_{t}\rangle- G_{nt}\sum_{k} \langle U_{nk}\rangle\langle V_{kt}\rangle$$

Individual specific random effects intercept ($\alpha_{it})$

For each individual $i$ and test $t$

Prior distribution: $p\left( \alpha_{it} \right)\sim N\left( 0, \psi_{t}^{2} \right)$

Variational distribution: $q\left( \alpha_{it} \right)\sim N(\mu_{\alpha_{it}},\sigma_{\alpha_{it}}^{2})$

Where updates are:

$$\sigma_{\alpha_{it}}^{2}=\left( \frac{\sum_{n} I\left[ n\in i \right]*1.0}{\langle\sigma_{t}^{2}\rangle}+\frac{1.0}{\psi_{t}^{2}} \right)^{-1}$$

$$\mu_{\alpha_{it}}={\sigma_{\alpha}^{2}}_{it}\sum_{n} \frac{I[n\in i]}{\left\langle\sigma_{t}^{2} \right\rangle}(r_{nt}^{\alpha_{it}})$$

$$r_{nt}^{\alpha_{it}}=Y_{nt}-{\langle\mu}_{t}\rangle-\sum_{l} X_{nl}\left\langle W_{lt} \right\rangle-G_{nt}{\langle F}_{t}\rangle- G_{nt}\sum_{k} \langle U_{nk}\rangle\langle V_{kt}\rangle$$

Latent context variance ($\gamma_{k}^{2})$

For each latent context $k$

Prior distribution: $p\left( 1/\gamma_{k}^{2} \right)\sim Gamma(\alpha_{0}, \beta_{0})$

Variational distribution: $q(1/\gamma_{k}^{2})\sim Gamma(\alpha_{\gamma_{k}^{2}}$,$\beta_{\gamma_{k}^{2}}$)

Where updates are:

$$\alpha_{\gamma_{k}^{2}}=\alpha_{0}+\frac{N}{2}$$

$$\beta_{\gamma_{k}^{2}}=\beta_{0}+\frac{\sum_{n} \langle U_{nk}^{2}\rangle}{2}$$

Random effects intercept variance ($\psi_{t}^{2})$

For each test $t$

Prior distribution: $p(1/\psi_{t}^{2})\sim Gamma(\alpha_{0}, \beta_{0})$

Variational distribution: $q\left( \frac{1}{\psi_{t}^{2}} \right)\sim Gamma\left( \alpha_{\psi_{t}^{2}}, \beta_{\psi_{t}^{2}} \right)$

Where updates are:

$$\alpha_{\psi_{t}^{2}}=\alpha_{0}+\frac{I}{2}$$

$$\beta_{\psi_{t}^{2}}=\beta_{0}+\frac{\sum_{i} \langle\alpha_{it}^{2}\rangle}{2}$$

Residual variance ($\sigma_{t}^{2})$

For each test $t$

Prior distribution: $p\left( 1/\sigma_{t}^{2} \right)\sim Gamma(\alpha_{0}, \beta_{0})$

Variational distribution: $q\left( \frac{1}{\sigma_{t}^{2}} \right)\sim Gamma(\alpha_{\sigma_{t}^{2}}, \beta_{\sigma_{t}^{2}})$

Where updates are:

$$\alpha_{\sigma_{t}^{2}}=\alpha_{0}+ \frac{N}{2}$$

$$\beta_{\sigma_{t}^{2}}=\beta_{0}+\frac{1}{2}\sum_{n} \left\langle\left( Y_{nt}-\mu_{t}-\sum_{i} I\left[ n\in i \right]{\langle\alpha}_{it}\rangle-\sum_{l} X_{nl}W_{lt}-G_{nt}F_{t}-G_{nt}\sum_{k} U_{nk}V_{kt} \right)^{2} \right\rangle$$

**SURGE coordinate ascent variational inference update algorithm**

Below, we provide pseudocode documenting the SURGE inference algorithm:

- Randomly initialize variational distributions $q\left( Z \right)$, where [$Z=$($F_{t}$, $V_{kt}$, $U_{nk}$, $\mu_{t}$, $W_{lt}$, $\alpha_{it}$, $\gamma_{k}^{2}$, $\psi_{t}^{2}$, $\sigma_{t}^{2}$)]
- iteration_num = 0
- While inference has not converged:
  - Update $q\left( U_{nk} \right) \forall n,k$
  - Update $q\left( V_{kt} \right) \forall k,t$
  - Update $q\left( \alpha_{it} \right) \forall i,t$
  - Update $q\left( C_{lt} \right) \forall l,t$
  - Update $q\left( F_{t} \right) \forall t$
  - If iteration_num >=5:
    - Update $\gamma_{k}^{2} \forall k$
  - Update $q\left( \psi_{t}^{2} \right) \forall t$
  - Update $q\left( \sigma_{t}^{2} \right) \forall t$
  - iteration_num=iteration_num+1
  - Converge if change in ELBO < $1e^{-2}$

**

*Fig S1: In simulation, we evaluated the runtime of SURGE latent context optimization across 10 independent runs on 1 core (top row) and interaction eQTL calling using LME4 given already-optimized SURGE latent contexts across 50 independent runs on 1 core (bottom row) as a function of eQTL sample size. The strength (variance) of the simulated interaction terms was set to .25, the number of SURGE latent contexts was set to 10, there were fixed effects from 20 covariates, and there were random effects due to sample repeat structure assuming 20 eQTL samples per individual.*

*Fig S2: In this simulation, we evaluate SURGE’s ability to re-capture simulated latent contexts as measured by the variance explained of the simulated components by the learned components (y-axis). In this simulation we vary the sample size (x-axis), the strength (variance) of the simulated interaction terms (colors), and the fraction of tests that are context-specific eQTLs for a particular context (A, B, C). For each parameter setting, we run 10 independent simulations. Each dot represents an independent simulation.*

*Fig S3: In this simulation, we evaluate SURGE’s ability to identify the number of simulated latent contexts (x-axis) over 10 independent simulations and SURGE optimizations (y-axis). In this simulation, the sample size was fixed to 250, the strength (variance) of the simulated interaction terms was set to .25 (A, B) and .5 (C, D), and the fraction of tests that are context-specific eQTLs for a particular context was set to .1 (A, C) and .3 (B,D).*

*Fig S4: (A) Proportion of expression variance explained (PVE; see Methods; y-axis) by the 20 SURGE latent contexts (x-axis) when SURGE was applied to samples concatenated across 10 GTEx v8 tissues. We show results for real data and data with permuted genotype (colors). The last 5 SURGE latent contexts were removed due to not passing our PVE filter of 1e-5 (see Methods). (B) Proportion of the SURGE-mediated expression variance explained (PSMVE; see methods; y-axis) by the 20 SURGE latent contexts (x-axis). The SURGE-mediated expression variance is the variance in gene expression explained by any of the SURGE latent contexts. Therefore, the points in this plot will necessarily sum to 1.*

*
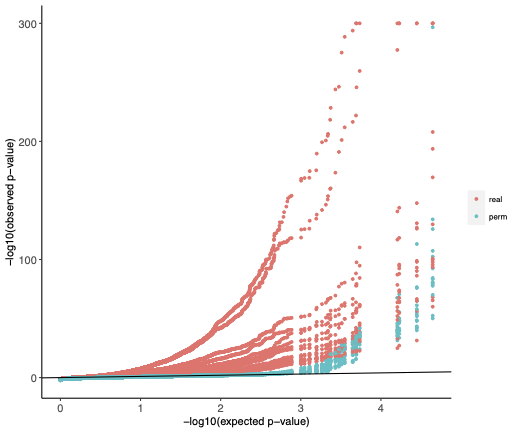
*

*Fig S5: Q-Q plot for 15 SURGE interaction eQTLs identified in GTEx v8 eQTL data aggregrated across 10 tissues. Red dots correspond to gene-level Bonferonni-corrected p-values for SURGE interaction-eQTLs relative to uniformly distributed p-values. Teal dots correspond to gene-level Bonferonni-corrected p-values from SURGE interaction eQTLs called with permuted genotype relative to uniformly distributed p-values.*

*
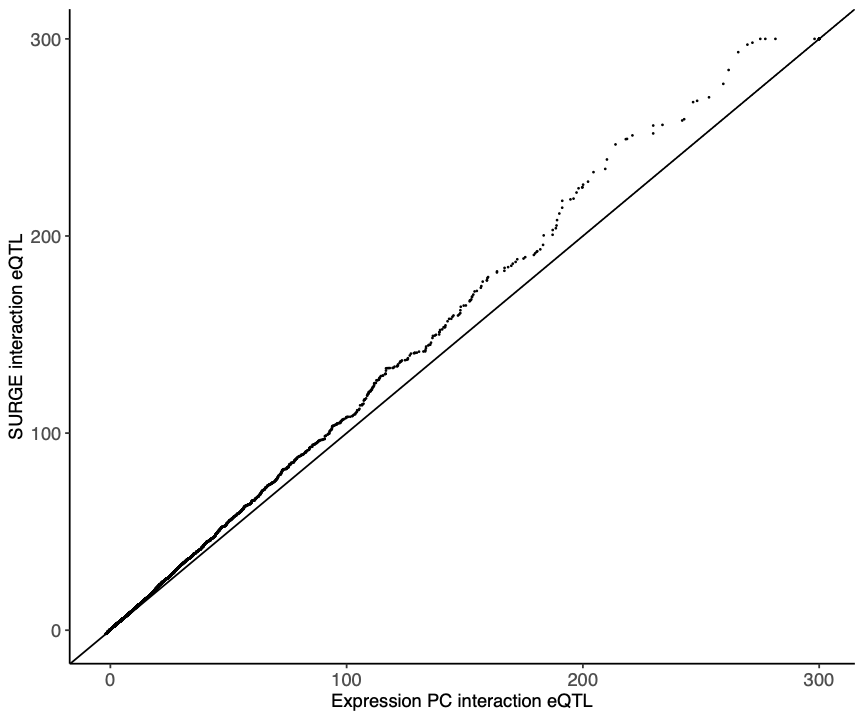
*

*Fig S6: Q-Q plot for SURGE interaction eQTLs (y-axis) identified in GTEx 10 tissue eQTL data relative to Expression PC interaction eQTLs (x-axis). 15 latent contexts and principal components were used for SURGE and Expression PC interaction eQTL analysis, respectively. We tested whether each variant-gene pair was significant for any of the latent contexts/expression PCs using a likelihood ratio test. Dots correspond to gene-level Bonferonni corrected p-values for SURGE interaction eQTLs (y-axis) and expression PC interaction eQTLs (x-axis).*

**

*Fig S7: (A) Absolute Pearson correlation between SURGE latent contexts identified in 10-tissue GTEx data without using a random effect intercept term during inference (y-axis) and with using a random effect intercept term during inference (x-axis). (B) Adjusted R-squared of each of the 15 SURGE latent contexts identified in the 10-tissue GTEx data without a random effect intercept explained by the 11 SURGE latent contexts identified in the 10-tissue GTEx data with a random effect intercept.*

**

*Fig S8: Scatter-plot of SURGE latent context 4 values (x-axis) by SURGE latent context 7 (y-axis) values across all GTEx version 8 samples concatenated over 10 GTEx tissues. Samples are colored by their loading on the first Genotype PC.*

*Fig S9: GTEx v8 RNA-seq samples are separated into 10 equally-sized bins according to their value on SURGE latent context 1, 2, 5, 6, and 7 (rows). The stacked bar plots depicts the average cell-type composition according to xCell estimates across all samples normalized to sum to 1(y-axis) in each of the 10 bins (x-axis). These results were generated when SURGE was applied to samples from 10 GTEx v8 tissues.*

*Fig S10: (A) Scatter-plot of SURGE latent context 5 values (x-axis) by xCell Epithelial cell-type enrichment scores (y-axis) for GTEx v8 samples from Colon Sigmoid, Small Intestine Terminal Ileum, and Stomach tissues. (B) Scatter-plot of SURGE latent context 6 values (x-axis) by xCell Neuron cell-type enrichment scores (y-axis) for GTEx v8 samples from Pituitary tissue.*

*Fig S11: Heatmap showing absolute Pearson correlation between the 8 SURGE latent contexts (identified when run on 10 GTEx tissues) (x-axis) and gene expression principal components and genotype principal components (y-axis).*

*Fig S12: These results were generated when SURGE was applied to samples from only Colon-Sigmoid GTEx v8 tissue. (A) GTEx v8 Colon-Sigmoid RNA-seq samples are separated into 10 equally-sized bins according to their value on SURGE latent context 1. The stacked bar plot depicts the average cell-type composition according to xCell enrichment scores across all samples normalized to sum to 1 (y-axis) in each of the 10 bins (x-axis). (B) We fit a multivariate linear model to predict SURGE latent context 1 from xCell cell type enrichment scores across 8 cell types. This plot shows the effect sizes and standard error of the effect sizes from this multivariate linear model (y-axis) for each of the 8 cell types that were used as fixed effects in the model (x-axis). 6 of the 7 cell types are predictive of SURGE latent context 1, even when conditioned on all other cell types.*


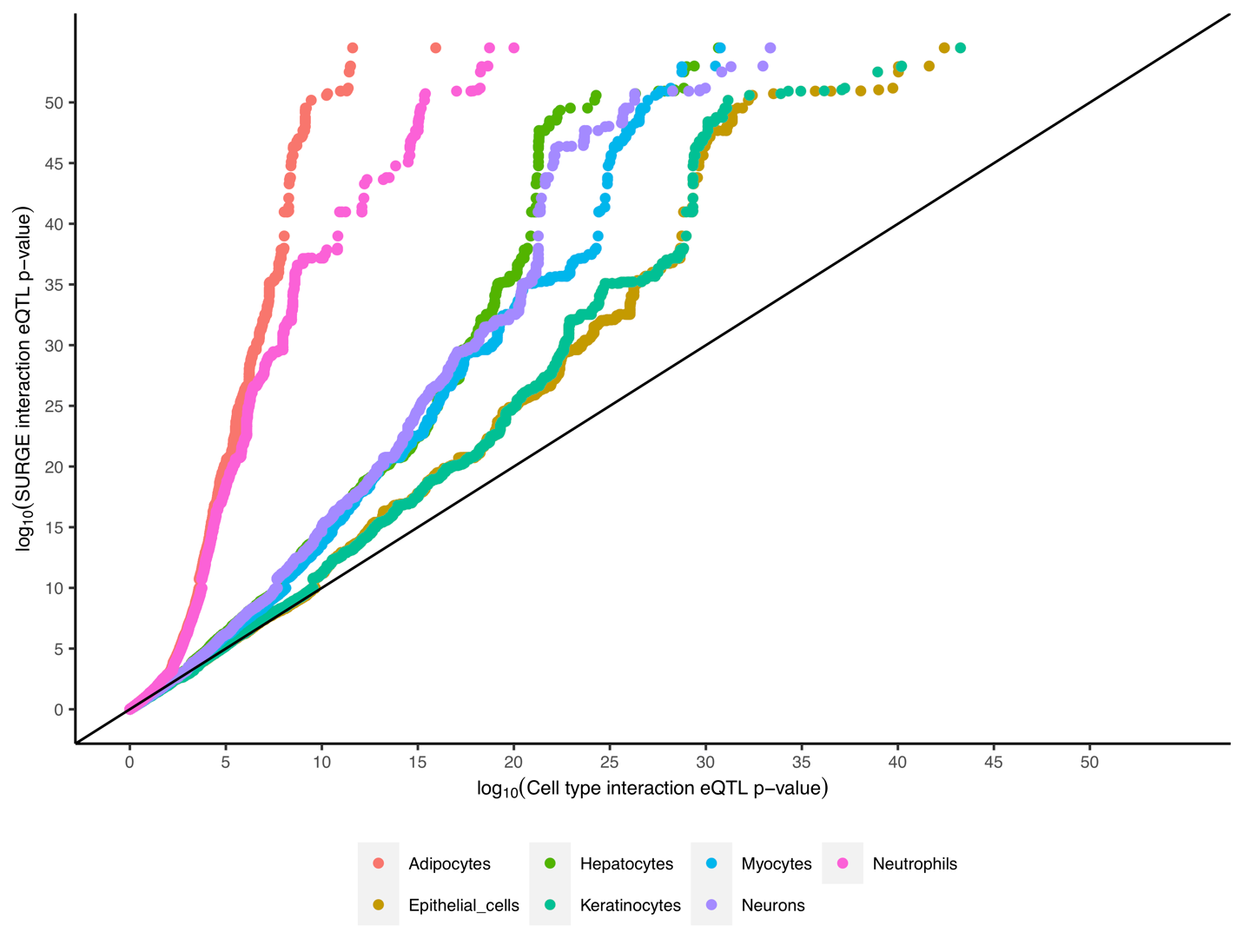


*Fig S13: These results were generated when SURGE was applied to samples from only Colon-Sigmoid GTEx v8 tissue. -log10(pvalues) of SURGE context 1 interaction eQTLs (y-axis) compared to -log10(pvalues) of interaction eQTLs using xCell cell type proportion from single cell type as the context (x-axis). Results shown for all 7 xCell cell types (colors).*

*Fig S14: Pseudocell aggregation of PBMC single cell expression data. (A). Distribution of number of cells (y-axis) per pseuodcell (x-axis). (B) Distribution of number pseudocells (y-axis) per individual (x-axis).*

**

*Fig S15: (A) Proportion of expression variance explained (PVE; see Methods; y-axis) by the 10 SURGE latent contexts (x-axis) when SURGE was applied to PBMC pseudocells. We show results for real data and data with permuted genotype (colors). The last 4 SURGE latent contexts were removed due to not passing our PVE filter of 1e-5 (see Methods). (B) Proportion of the SURGE-mediated expression variance explained (PSMVE; see methods; y-axis) by the 10 SURGE latent contexts (x-axis). The SURGE-mediated expression variance is the variance in gene expression explained by any of the SURGE latent contexts. Therefore, the points in this plot will necessarily sum to 1.*

*
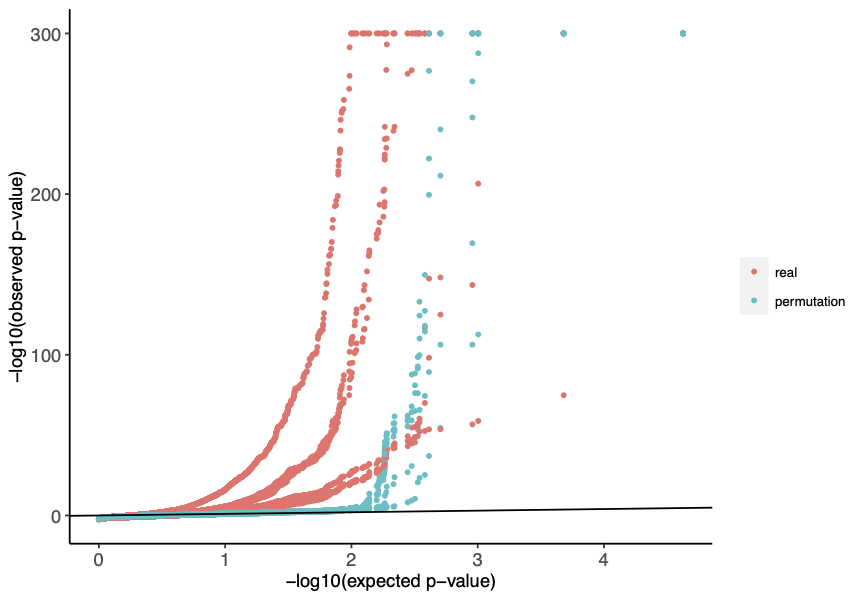
*

*Fig S16: Q-Q plot for 6 SURGE interaction eQTLs identified in PBMC single-cell eQTL data. Red dots correspond to gene-level Bonferonni-corrected p-values for SURGE interaction-eQTLs relative to uniformly distributed p-values. Teal dots correspond to gene-level Bonferonni-corrected p-values from SURGE interaction eQTLs called with permuted genotype relative to uniformly distributed p-values.*

**

*Fig S17: Q-Q plot for SURGE interaction eQTLs (y-axis) identified in PBMC single-cell eQTL data relative to Expression PC interaction eQTLs (x-axis). 6 latent contexts and principal components were used for SURGE and Expression PC interaction eQTL analysis, respectively. We tested whether each variant-gene pair was significant for any of the latent contexts/expression PCs using a likelihood ratio test. Dots correspond to gene-level Bonferonni corrected p-values for SURGE interaction eQTLs (y-axis) and expression PC interaction eQTLs (x-axis).*

**

*Fig S18: SURGE latent context loadings of pseudocells (y-axis) stratified by cell type according to marker gene expression profiles for each of the 6 identified SURGE latent contexts. Results shown for both (A) cell-types and (B) fine-resolution cell-types.*

*
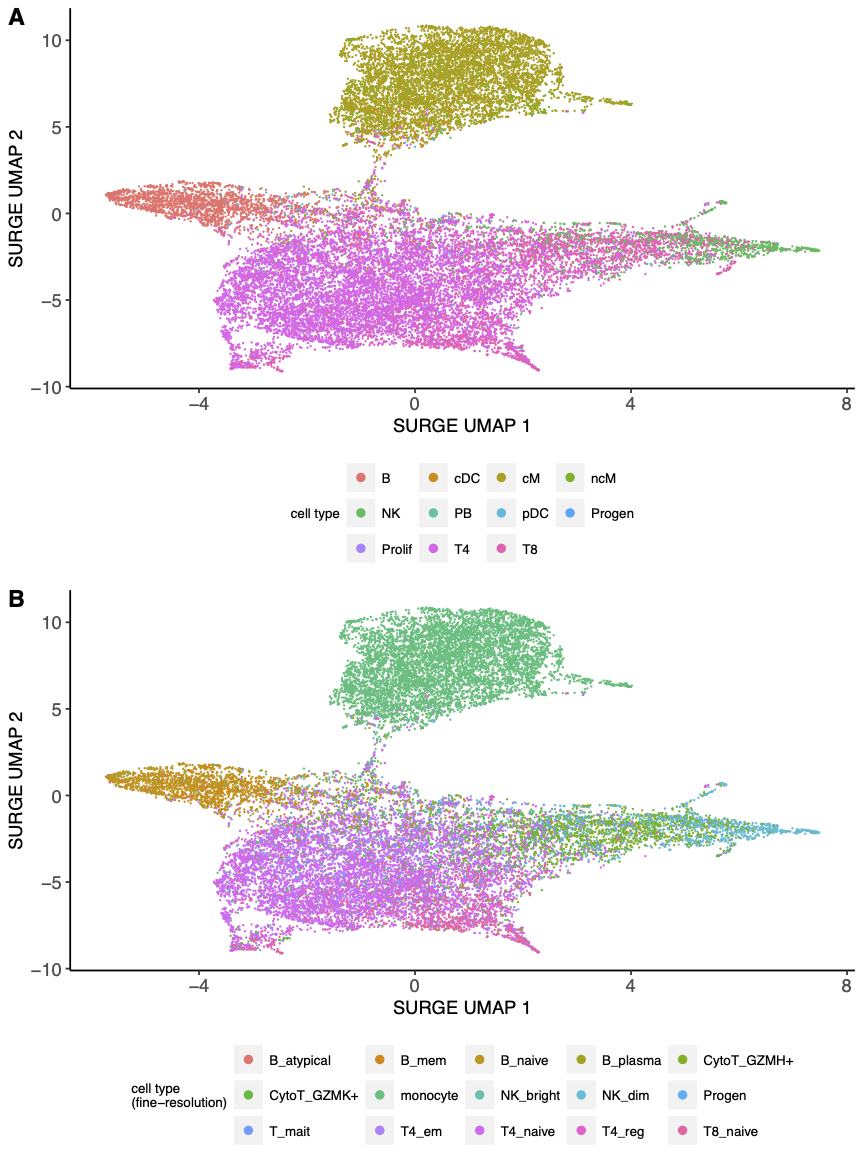
*

*Fig S19: UMAP-projected SURGE latent context loadings of pseudocells (x and y-axis) colored by (A) cell-types and (B) fine-resolution cell-types.*

*
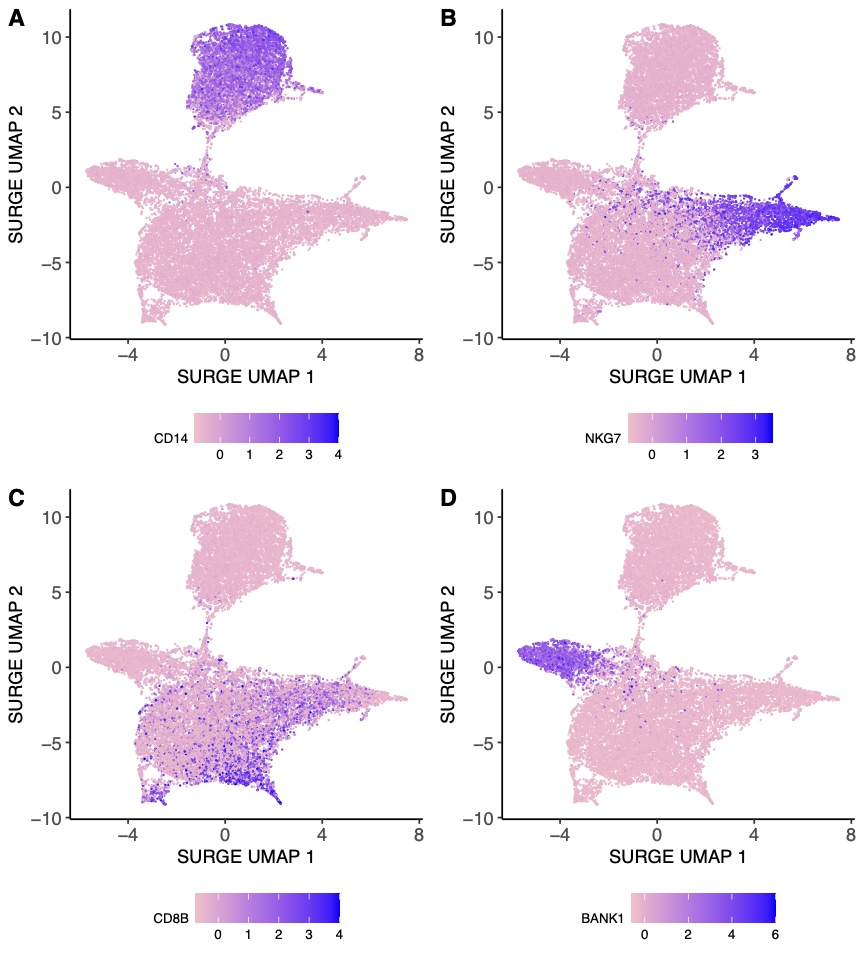
*

*Fig S20: UMAP-projected SURGE latent context loadings of pseudocells (x and y-axis) colored by expression levels of four marker genes: (A) CD14, (B) NKG7, (C) CD8B, (D) BANK1. CD14 is a marker for monocytes, NKG7 is a maker for NK cells, CD8B is a marker for T cells and BANK1 is a marker for B cells.*

**

*Fig S21: Density of (y-axis) SURGE latent context 1 loadings (x-axis) on pseudocells annotated as monocytes according to marker-gene expression profiles color-stratified by disease status of individuals corresponding to pseudocells.*

**

*Fig S22: Heatmap showing variance explained of each of the gene expression principal components (x-axis) by the 6 SURGE latent contexts (y-axis) (identified when run on PBMC single-cell eQTL data set).*

*Fig S23: Heatmap showing variance explained of each of the six SURGE latent contexts (y-axis) by various pseudocell sample characteristics.*

**

*Fig S24: Number of colocalizations identified (PPH4 > .95; y-axis) between 15 independent GWAS studies (x-axis) and various categories of eQTLs called from pseudocells.*

**

*Fig S25: Number of colocalizations identified (PPH4 > .95; y-axis) between 15 independent GWAS studies (x-axis) and SURGE interaction eQTLs (green) and expression PC interaction eQTLs (grey). Results stratified by latent context number of expression PC number for SURGE or Expression PC interaction eQTLs, respectively.*

**

Fig S26: S-LDSC enrichment (y-axis) of squared standard eQTL effect sizes (black line) and SURGE predicted squared eQTL effect size at a specific SURGE latent context value (pink line at a specific x-axis position) meta-analyzed across blood-related traits shown for 6 SURGE latent contexts. SURGE predicted eQTL effect sizes at a particular SURGE latent context value was calculated at 200 equally spaced positions along the range of SURGE latent context values. Black dashed line represents 95% confidence on the standard eQTL S-LDSC enrichment. Light pink region depects 95% confidence on the SURGE predicted eQTL S-LDSC enrichment. Trait category of “blood” consists of GWAS for eosinophil count, reticulocyte count, lymphocyte count, corpuscular hemoglobin, monocyte count, platelet count, blood platelet volume, red blood count, and white blood count.

| **SURGE latent context** | **Number of genes with SURGE interaction-eQTL**  **(per context eFDR < .05)** | **Number of genes with with SURGE interaction-eQTL**  **(per context eFDR < .1)** |
| --- | --- | --- |
| 1 | 5649 | 6771 |
| 2 | 5336 | 6335 |
| 3 | 2363 | 3223 |
| 4 | 4261 | 5677 |
| 5 | 1311 | 1872 |
| 6 | 985 | 1672 |
| 7 | 1087 | 1682 |
| 8 | 587 | 1187 |
| 9 | 0 | 749 |
| 10 | 0 | 644 |
| 11 | 0 | 622 |
| 12 | 0 | 0 |
| 13 | 0 | 349 |
| 14 | 0 | 1153 |
| 15 | 0 | 2 |

*Table S1: The number of genes with a genome-wide significant variant that is a SURGE interaction-eQTL for each of the 15 SURGE latent contexts (rows) identified when SURGE was run on 10 GTEx tissues. Significance determined via “per context empirical FDR” (eFDR) correction according to an empirical null distribution generated from a permutation analysis (see Methods).*

| **SURGE latent context** | **Number of genes with SURGE interaction-eQTL**  **(all context eFDR < .05)** | **Number of genes with with SURGE interaction-eQTL**  **(all context eFDR < .1)** |
| --- | --- | --- |
| 1 | 4386 | 5427 |
| 2 | 4234 | 5175 |
| 3 | 2118 | 2926 |
| 4 | 3225 | 4337 |
| 5 | 1418 | 2032 |
| 6 | 1243 | 1900 |
| 7 | 1282 | 1896 |
| 8 | 1019 | 1526 |
| 9 | 768 | 1289 |
| 10 | 746 | 1380 |
| 11 | 730 | 1379 |
| 12 | 608 | 1108 |
| 13 | 676 | 1302 |
| 14 | 938 | 1685 |
| 15 | 479 | 982 |

*Table S2: The number of genes with a genome-wide significant variant that is a SURGE interaction-eQTL for each of the 15 SURGE latent contexts (rows) identified when SURGE was run on 10 GTEx tissues. Significance determined via “all context empirical FDR” (eFDR) correction according to an empirical null distribution generated from a permutation analysis (see Methods).*

| **SURGE latent context** | **Pvalue of association between SURGE latent context and tissue identity** | **Adjusted R-squared** |
| --- | --- | --- |
| 1 | <2.2e-16 | 0.850 |
| 2 | <2.2e-16 | 0.877 |
| 3 | <2.2e-16 | 0.619 |
| 4 | <2.2e-16 | 0.152 |
| 5 | <2.2e-16 | 0.510 |
| 6 | < 2.2e-16 | 0.540 |
| 7 | < 2.2e-16 | 0.473 |
| 8 | < 2.2e-16 | 0.392 |
| 9 | < 2.2e-16 | 0.172 |
| 10 | < 2.2e-16 | 0.068 |
| 11 | < 2.2e-16 | 0.086 |
| 12 | < 2.2e-16 | 0.059 |
| 13 | < 2.2e-16 | 0.070 |
| 14 | < 2.2e-16 | 0.051 |
| 15 | 2.61e-8 | 0.011 |

*Table S3: For each SURGE latent factor we regressed tissue identity (categorical variable) onto the SURGE latent context. We report the p-value from F-statistic and the adjusted R-squared.*

| **SURGE latent context** | **Pvalue of association between SURGE latent context and known ancestry** | **Adjusted R-squared** |
| --- | --- | --- |
| 1 | 1.73e-6 | 0.00675 |
| 2 | 0.394 | 2.24e-5 |
| 3 | <2.2e-16 | 0.222 |
| 4 | <2.2e-16 | 0.417 |
| 5 | 1.49e-10 | 0.0114 |
| 6 | 3.72e-16 | 0.0177 |
| 7 | <2.2e-16 | 0.0709 |
| 8 | <2.2e-16 | 0.0219 |
| 9 | 1.17e-10 | 0.0115 |
| 10 | <2.2e-16 | 0.00224 |
| 11 | 0.224 | 0.000404 |
| 12 | 0.100 | 0.000907 |
| 13 | 1.20e-8 | 0.00925 |
| 14 | 0.0455 | 0.00137 |
| 15 | 0.000902 | 0.00352 |

*Table S4: For each SURGE latent factor we regressed known ancestry (categorical variable) onto the SURGE latent context. We report the p-value from F-statistic and the adjusted R-squared.*

| **SURGE latent context** | **Pvalue of association between SURGE latent context and xCell cell type enrichment scores** | **Adjusted R-squared** |
| --- | --- | --- |
| 1 | 1.73e-6 | 0.785 |
| 2 | 0.394 | 0.854 |
| 3 | <2.2e-16 | 0.1403 |
| 4 | <2.2e-16 | 0.0821 |
| 5 | 1.49e-10 | 0.300 |
| 6 | 3.72e-16 | 0.431 |
| 7 | <2.2e-16 | 0.169 |
| 8 | <2.2e-16 | 0.215 |
| 9 | <2.2e-16 | 0.0964 |
| 10 | <2.2e-16 | 0.0413 |
| 11 | <2.2e-16 | 0.1043 |
| 12 | <2.2e-16 | 0.0464 |
| 13 | <2.2e-16 | 0.0386 |
| 14 | <2.2e-16 | 0.0270 |
| 15 | 1.39e-5 | 0.00658 |

*Table S5: For each SURGE latent factor we regressed xCell cell type enrichment estimates from 7 cell types (Adipocytes, Epithelial cells, Hepatocytes, Keratinocytes, Monocytes, Neurons, and Neutrophils) onto the SURGE latent context. We report the p-value from F-statistic and the adjusted R-squared.*

| **SURGE latent context** | **Number of genes with SURGE interaction-eQTL**  **(per context eFDR < .05)** | **Number of genes with with SURGE interaction-eQTL**  **(per context eFDR < .1)** |
| --- | --- | --- |
| 1 | 1407 | 1641 |
| 2 | 0 | 657 |
| 3 | 0 | 0 |
| 4 | 514 | 824 |
| 5 | 0 | 297 |
| 6 | 0 | 406 |

*Table S6: The number of genes with a genome-wide significant variant that is a SURGE interaction-eQTL for each of the 6 SURGE latent contexts (rows) identified when SURGE was run on PBMC single cell eQTL data. Significance determined via “per context empirical FDR” (eFDR) correction according to an empirical null distribution generated from a permutation analysis (see Methods).*

| **SURGE latent context** | **Number of genes with SURGE interaction-eQTL**  **(all context eFDR < .05)** | **Number of genes with with SURGE interaction-eQTL**  **(all context eFDR < .1)** |
| --- | --- | --- |
| 1 | 0 | 1403 |
| 2 | 0 | 672 |
| 3 | 0 | 374 |
| 4 | 0 | 793 |
| 5 | 0 | 421 |
| 6 | 0 | 530 |

*Table S7: The number of genes with a genome-wide significant variant that is a SURGE interaction-eQTL for each of the 6 SURGE latent contexts (rows) identified when SURGE was run on PBMC single cell eQTL data. Significance determined via “all context empirical FDR” (eFDR) correction according to an empirical null distribution generated from a permutation analysis (see Methods).*

| **SURGE latent context** | **Pvalue of association between SURGE latent context and cell type** | **Adjusted R-squared** |
| --- | --- | --- |
| 1 | <2.2e-16 | 0.838 |
| 2 | <2.2e-16 | 0.424 |
| 3 | <2.2e-16 | 0.049 |
| 4 | <2.2e-16 | 0.542 |
| 5 | <2.2e-16 | 0.025 |
| 6 | <2.2e-16 | 0.048 |

*Table S8: For each SURGE latent factor we regressed known cell type (categorical variable; T8, B, cDC, cM, ncM, NK, PB, pDC, Progen, Prolif, T4, T8) onto the SURGE latent context. We report the p-value from F-statistic and the adjusted R-squared.*

| **SURGE latent context** | **Pvalue of association between SURGE latent context and fine-resolution cell type** | **Adjusted R-squared** |
| --- | --- | --- |
| 1 | <2.2e-16 | 0.763 |
| 2 | <2.2e-16 | 0.532 |
| 3 | <2.2e-16 | 0.0978 |
| 4 | <2.2e-16 | 0.537 |
| 5 | <2.2e-16 | 0.010 |
| 6 | <2.2e-16 | 0.070 |

*Table S9: For each SURGE latent factor we regressed known fine-grained cell type labels (categorical variable; B_mem, B_naive, B_Plasma, CytoT_GZMH+, CytoT_GZMK+, monocyte, NK_bright, NK_dim, Progen, T_mait, T4_em, T4_naive, T4_reg, T8_naive) onto the SURGE latent context. We report the p-value from F-statistic and the adjusted R-squared.*

| ***Hallmark gene set*** | ***Latent context 5*** | ***Latent context 6*** |
| --- | --- | --- |
| *Interferon gamma response* | *4.40e-12* | *NS* |
| *Interferon alpha response* | *4.19e-5* | *NS* |
| *IL2 Stat5 signaling* | *.00409* | *NS* |
| *Complement* | *.00600* | *NS* |
| *Hypoxia* | *.00654* | *NS* |
| *Coagulation* | *.0408* | *NS* |
| *Allograft rejection* | *NS* | *0.00247* |

*Table S10: Bonferroni corrected p-values (Fisher’s exact) from gene set enrichment of genes strongly correlated with Latent Context 5 and 6 (columns) within Hallmark gene sets (rows). Only gene sets with significant enrichment (Bonferroni p-value <= .05) in genes strongly correlated with latent context 5 or latent context 6 are shown. NS means not significant (Bonferroni p-value > .05).*

| ***MSigDB Biological Process gene set*** | ***Latent context 5*** | ***Latent context 6*** |
| --- | --- | --- |
| *Translation* | *NS* | *3.48e-7* |
| *Cellular biosynthetic process* | *NS* | *2.44e-5* |
| *Biosynthetic process* | *NS* | *8.29e-5* |
| *Cell structure disassembly during apoptosis* | *NS* | *.0308* |
| *Immune response* | *NS* | *.0377* |
| *Immune system process* | *NS* | *.0430* |

*Table S11: Bonferroni corrected p-values (Fisher’s exact) from gene set enrichment of genes strongly correlated with Latent Context 5 and 6 (columns) within MSigDB Biological process gene sets (rows; c5.bp.v5.1). Only gene sets with significant enrichment (Bonferroni p-value <= .05) in genes strongly correlated with latent context 5 or latent context 6 are shown. NS means not significant (Bonferroni p-value > .05).*
