# Supplementary material for: SURGE: uncovering context-specific genetic-regulation of gene expression from single-cell RNA sequencing using latent-factor models
Source: Genome Biol. 2024 Jan 22;25:28. doi: 10.1186/s13059-023-03152-z (PMC10801966; doi:10.1186/s13059-023-03152-z)
Supplement: Supplementary file 2 — Additional file 2. Review history. [file 13059_2023_3152_MOESM2_ESM.docx]

**Round 1**

**Reviewer 1**

The manuscript by Strober and colleagues presents a new computational method: SURGE (Single-cell Unsupervised Regulation of Gene Expression), to discover context-eQTLs in scRNA-seq data, without pre-specifying the contexts. This method uses matrix factorization to learn a continuous representation of the cellular contexts that modulate the genetic effects. The authors present application to simulated data to show the performance of the model and also to real scRNA-seq data from PBMCs derived from 224 donors.

Specific comments are provided below:

Major comments:
Once SURGE identifies the latent contexts, there is no explicit quantification of each latent context. The authors describe that their model has successfully detected the ancestry effect in latent contexts 4 and 7 in Figure 2B, while no explicit quantification or validation is provided for this claim. How is the robustness/significance of each latent context quantified?

The manuscript uses S-LDSC as a means to provide support to their findings. One major problem with the manuscript is the lack of validation and cross-comparison with previously described and widely used tools in context-eQTL mapping such as Eagle, MashR, etc.

The manuscript should address the novelty/advantage of SURGE compared to similar approaches such as those based on PCA and the one developed by Zhernakova et al, 2017. Furthermore, how does SURGE compare to an approach that first identifies latent contexts and then performs context-eQTL mapping?

To provide stronger support and applicability of this model to broad unknown contexts, it would also be worthwhile to explore other known contexts besides cell type differentiation and variation. For example in the sceQTL application, what is captured by latent variable 4 and 5? Are they interpretable? More generally one important limitation of the manuscript in the current form is that it doesn't show the biological significance of the latent contexts

The discussion reads quite redundantly, with limited critical discussion in terms of best applications, limitations etc. For example, would SURGE perform well with contexts that have small effects or that are highly correlated with each other?

Minor comments:
The method focuses on the most significant variant for each eGene, identified by standard eQTL mapping analysis. The manuscript should discuss potential limitations due to multiple independent eQTLs known for a large number of genes.

The manuscript should discuss any limitation due to the fact that the model does not consider unknown contexts in parallel, but rather sequentially.

**Reviewer 2**

Strober et al present an systematic platform for interaction QTL testing. While the method is elegant there is little comparison to previous methods / setups showing the benefit of this all encompassing work.

Major:
The paper shows an interesting end to end model for interaction QTL mapping. However a comparison to existing methods such as CellRegMap / Struct LMM (Stegle Lab), or methods like used in the Nathan et al paper NG are missing.
What is the gain here?

Could the authors add more information on the FDR procedure? How did th authors controlled for testing multiple genes / multiple context per genes?
Linked what is meant with: "independent variant-gene pairs", it seems like genes could still be linked?

Is the SURGE model fitted with the fixed effects included?

How did the authors arrive at a PVE > 1e-5 a a cutt off?

What is the run time of this model, also in the complete mode (i.e. not the fitting step)?

Minor:
Why were 80 factors corrected out of the expression data in GTEx wouldn't this remove a lot of contexts?

For the GTEx analysis did the authors also include a random effect term for the eQTL mapping in GTEx? in the SC analysis it is specifically mentioned but not for the other analysis.


Please consider the following questions:
- Are the methods appropriate to the aims of the study, are they well described, and are necessary controls included?
Yes
- Are the conclusions adequately supported by the data shown?
Yes, but a comparison between methods would be good. What is the benefit of this method over methods by Cuomo et al and Nathan et al, or even the methods highlighted by the authors (Vochterloo et al).
- Are sufficient details provided to allow replication and comparison with related analyses that may have been performed?
Yes
- Is the method likely to be of broad utility? Is any software component easy to install and use? Please indicate briefly the novel features and/or advantages of the method, and/or please reference the relevant publications and which methods, if any, it should be compared with.
Yes, but novelty / applicability over existing methods is not clear.
- Is the paper of broad interest to others in the field, or of outstanding interest to a broad audience of biologists?
yes

**Authors’ response**

*We thank both reviewers for the detailed comments and suggestions. We addressed each comment, as described below, and we include all results in the revised paper.*

*Independent from the suggested revisions, we have corrected an analysis error related to the number of SURGE latent factors identified in both the GTEx (10 tissue) and the single cell eQTL analysis. Our error was caused by an outdated filter on latent contexts that removed latent contexts with too few interaction eQTLs after our permutation-based multiple testing correction. Specifically, we previously identified 8 and 5 SURGE latent factors for the GTEx and single cell analyses, respectively. After correcting the mistake, we now discovered 15 and 6 SURGE latent factors, respectively. All previous SURGE latent factors are re-discovered in the updated analysis (ie. the updated SURGE latent factors are a superset of the previous SURGE latent factors). We have updated all text, figures, and tables accordingly. We do not believe this correction has affected any claims made in the paper as the top SURGE latent factors in each analysis remain unchanged, we simply have additional SURGE latent contexts to explore per data set.*

**Reviewer 1**

1. Once SURGE identifies the latent contexts, there is no explicit quantification of each latent context. The authors describe that their model has successfully detected the ancestry effect in latent contexts 4 and 7 in Figure 2B, while no explicit quantification or validation is provided for this claim. How is the robustness/significance of each latent context quantified?

*We thank the reviewer for this comment. We have added the following sentence to the third results paragraph in order to clarify how SURGE latent contexts can be understood:*

*“After inference, SURGE latent contexts can be associated with available measurements and annotations to help interpret their biological meaning.”*

*We have also updated the main text to include the corresponding association statistics for each discussed connection between SURGE latent context and measurement annotation. This includes five new supplementary tables that summarize the associations between SURGE latent contexts and GTEx tissue identity (Table S3), GTEx known ancestry (Table S4), GTEx xCell estimates (Table S5), and single-cell cell-types (Table S8 and Table S9).*

2. The manuscript uses S-LDSC as a means to provide support to their findings. One major problem with the manuscript is the lack of validation and cross-comparison with previously described and widely used tools in context-eQTL mapping such as Eagle, MashR, etc.

*We agree it is important to compare SURGE to existing interaction eQTL calling approaches. We have updated the manuscript to include an extensive comparison of SURGE interaction eQTLs with interaction eQTLs using pre-defined expression PCA contexts (see response comment 3 below for more details). However, we do not include a comparison with Eagle as it relies on allele-specific expression data which cannot be extracted from single-cell 10X sequencing data and therefore is not applicable to most single cell eQTL data sets, and ASE is out of scope of this paper. We also do not include a comparison to MashR. MashR has very different goals and outputs relative to SURGE making it not directly comparable. Most notably, MashR does not seek to identify interaction-eQTLs (genetic variants with significantly different effects on gene expression between contexts). Instead, MashR robustly infers standard eQTL effects by sharing information across pre-defined discrete contexts. Interaction eQTLs (eg from SURGE) and standard eQTLs (eg from MashR) represent two distinct sources of information describing different types of effects of a genetic variant on gene expression.*

3. The manuscript should address the novelty/advantage of SURGE compared to similar approaches such as those based on PCA and the one developed by Zhernakova et al, 2017. Furthermore, how does SURGE compare to an approach that first identifies latent contexts and then performs context-eQTL mapping?

*We appreciate this suggestion as we believe the paper is stronger with the addition of a comparison to existing eQTL calling approaches.*

*When SURGE is run on an eQTL data set, it produces two distinct outputs. The first is the SURGE latent contexts, or continuous sample-level representations whose linear interaction with genotype explains the most variation in gene expression levels. The second is the set of specific interaction eQTLs, or variant-gene pairs whose linear interaction between genotype and the inferred SURGE latent contexts significantly affects gene expression levels. The SURGE latent contexts alone provide unique information describing properties of the eQTL data set that is not provided by standard approaches for interaction eQTL calling. Specifically, the SURGE latent contexts represent the dominant axes of genetic regulation of gene expression defining an eQTL data set. This can be viewed analogously to the utility of PCA to uncover the dominant axes of gene expression variation in a gene expression data set. However, we agree with the reviewer in that the manuscript will be strengthened by explicit comparisons between SURGE interaction eQTLs and interaction eQTLs identified by existing approaches.*

*In our original manuscript, the paper included a supplementary figure (previously Fig. S10, currently Fig. S13) demonstrating that SURGE is higher powered to detect interaction eQTLs relative to the approach presented in Kim-Hellmuth et al. Science 2020, which tests the interaction between genotype and cell-type enrichment score estimates (quantified via xCell) on gene expression.*

*During the revision, we have updated the manuscript to include analysis demonstrating the benefits of SURGE interaction eQTLs over expression PCA-based interaction eQTLs (that is, interactions between gene expression principal components and genotype on gene expression). CellRegMap (Cuomo et al. Molecular Systems Biology 2022) and the method developed in Nathans et al. Nature 2022 are examples methods developed to test for expression PC interaction eQTLs. We used to use our own implementation of expression PC interaction eQTL calling in order to minimize modeling assumption differences with SURGE and allow a focused comparison between the use of SURGE latent contexts and expression PCs in interaction eQTL calling. We have added text to the discussion specifying limitations of these modeling assumptions (see response to Reviewer 1, comment 5 below), but believe this choice is orthogonal to the advances made by SURGE. We show that, in both the GTEx eQTL analysis and single cell PBMC eQTL analysis, SURGE latent contexts identify slightly more interaction eQTLs than expression PCs (Fig. S6 and Fig. S17). We attribute the fairly concordant number of identified interaction eQTLs to the high levels of correlation between the top SURGE latent contexts and expression PCs (Fig. S11 and Fig. S22) and the regularization (ie. the ARD prior, see methods section “SURGE model overview” for more details) placed on the SURGE latent contexts. Importantly however, the eQTLs identified are not identical, and we show that SURGE interaction eQTLs identify significantly more GWAS trait colocalizations than expression PC interaction eQTLs. This analysis and its main results are summarized in an updated main figure panel (Fig. 3C), a new supplementary figure (Fig. S25), and the following excerpt from the main results text:*

*“In addition, we compared the number of trait colocalizations identified using SURGE interaction eQTLs with expression PC interaction eQTLs (see Methods). Despite both methods identifying comparable numbers of interaction eQTLs (Fig S17), SURGE interaction eQTLs colocalized (PPH4 > .95) with 2.4 more loci per trait on average across 15 independent traits (Fig. 3C; p < 0.017, paired Wilcoxon rank sum test). SURGE identified 66 trait colocalizations (PPH4 > .95) that could not be replicated (PPH4 > .1) by the expression PC interaction eQTL analysis, whereas the expression PC interaction eQTL only identified 31 trait colocalizations not replicated by SURGE interaction eQTLs. Notably, 50 of the 66 trait colocalizations unique to SURGE were discovered in the SURGE latent contexts not well explained by expression PCs (latent contexts 3, 5 and 6; Fig. S18, Fig. S25) demonstrating the importance of not relying exclusively on expression PCs for interaction eQTL calling.”*

*Lastly, we attempted to compare SURGE with the method developed in Zhernakova et al. Nat. Genet. 2017, but could not get their software to run without crashing. We contacted the authors and they informed us they are no longer actively maintaining the software. Conceptually however, the method developed in Zhernakova et al. Nat. Genet. 2017 is limited to discovering contexts explained perfectly by the expression measurements of a single gene. Alternatively, SURGE can uncover any continuous-valued latent context (including latent contexts not explained by gene expression) and is therefore capable of uncovering a broader set of interaction eQTLs than the method developed in Zhernakova et al. Nat. Get. 2017.*

4. To provide stronger support and applicability of this model to broad unknown contexts, it would also be worthwhile to explore other known contexts besides cell type differentiation and variation. For example in the sceQTL application, what is captured by latent variable 4 and 5? Are they interpretable? More generally one important limitation of the manuscript in the current form is that it doesn't show the biological significance of the latent contexts

*We attempted to identify the biological mechanism responsible for each of the inferred SURGE latent contexts. In the single-cell eQTL analysis, each of the SURGE latent contexts was associated with all available pseudocell sample characteristics including cell type composition, age of donor, sex of donor, ancestry of donor, whether the donor had SLE, estimated ISG score (see Perez et al. Science 2022), and various technical covariates related to 10X sequencing. This new analysis is summarized in the new supplementary figure S23. Combined with previous analysis (Fig. S22), we are unable to uncover the biological mechanism underlying SURGE latent contexts 3, 5, and 6. While each of these contexts is significantly associated with cell type (Table S8, S9), only 4.9%, 2.5%, and 4.8% of the latent context variance can be explained by cell type, respectively; meaning these contexts contain a great deal of additional information other than cell type. However, we do not view this as a failure, and in fact view it as one of the benefits of an unsupervised approach to eQTL calling. SURGE uncovers a dominant axis of eQTL effects within the single cell eQTL data set that would be missed using a more conventional approach to eQTL calling (ie. where the interaction contexts were set to be expression PCs or known cell types). Importantly, even though we do not fully understand the biological mechanism underlying these latent contexts, we show interaction eQTLs corresponding to these contexts colocalize with many trait and disease loci that would have otherwise been missed (Figure S25). Future researchers may uncover the biological meaning of some contexts through specialized analysis and even experimental follow-up beyond our domain expertise, similar to work that has led to understanding PCs for gene expression data in other projects. Overall, this comment addresses a feature of all unsupervised approaches that can be viewed both as an advantage for novel discovery and a challenge for interpretation.*

5. The discussion reads quite redundantly, with limited critical discussion in terms of best applications, limitations etc. For example, would SURGE perform well with contexts that have small effects or that are highly correlated with each other?

*We have greatly revised the discussion. It now contains the following paragraph discussing the limitations of SURGE (including a point on power to detect different classes of latent contexts):*

*Although SURGE allows for identification of context-specific eQTLs without pre-specifying contexts, it has several limitations. First, SURGE is limited to discovery of interaction eQTLs from contexts whose effects explain a large fraction of gene expression variation aggregated across many genes in the genome (Fig. 1C) and will be underpowered to detect interaction eQTLs with small effect sizes or those from contexts that interact with the genetic regulation gene expression in a small number of genes. Second and like many unsupervised algorithms, it can be challenging to interpret the biological meaning of some SURGE latent contexts, particularly those that capture signals independent of observed measurement annotations such as cell type or sample covariates. Third, SURGE latent context inference and SURGE interaction eQTL calling is limited to the most significant variant for each eGene for each context, hindering discovery of multiple independent context specific eQTLs regulating a single gene in a single context. Fourth, SURGE makes the simplifying assumption that the residual variance in gene expression is distributed normally. Recent work has demonstrated the benefits of non-normal distributions in single cell interaction eQTL calling [13]. We leave the extension of SURGE with alternative residual distributions to future work. Fifth, SURGE cannot be reasonably scaled to more than 50,000 expression samples (Fig. S1). This does not present a concern for existing eQTL data sets, however, SURGE optimization could be extended in the future to scale to arbitrarily large eQTL data sets using parallel programming or stochastic optimization [37].*

6. The method focuses on the most significant variant for each eGene, identified by standard eQTL mapping analysis. The manuscript should discuss potential limitations due to multiple independent eQTLs known for a large number of genes.

*The third limitation described in the updated discussion text (see comment 5 response) acknowledges this concern. To our knowledge, no existing work has identified multiple, independent interaction-eQTLs per gene despite the large number of papers devoted to identifying interaction eQTLs. We leave this to future work.*

7. The manuscript should discuss any limitation due to the fact that the model does not consider unknown contexts in parallel, but rather sequentially.

*We would like to clarify that all SURGE latent contexts (and corresponding SURGE interaction eQTL effect sizes) are jointly fit to on gene expression. Combined with the Automatic Relevance Determination priors placed on the SURGE latent contexts, this will encourage each SURGE latent context to capture unique signals and enhance the interpretability of the contexts.*

**Reviewer 2**

1. The paper shows an interesting end to end model for interaction QTL mapping. However a comparison to existing methods such as CellRegMap / Struct LMM (Stegle Lab), or methods like used in the Nathan et al paper NG are missing.
What is the gain here?

*We thank the reviewer for the opportunity to clarify the advantages offered by our method. Please refer to our response to comment 2 and 3 by reviewer 1.*

2. Could the authors add more information on the FDR procedure? How did the authors controlled for testing multiple genes / multiple context per genes?
Linked what is meant with: "independent variant-gene pairs", it seems like genes could still be linked?

*First, we will describe what was meant by “independent variant-gene pairs”. To reiterate, SURGE inference consists of two distinct steps. In the first step, SURGE uses variational inference to infer SURGE latent contexts using expression and genotype data from a subset of variant-gene pairs. As a part of the inference procedure in the first step, SURGE infers the SURGE interaction eQTL effect sizes, but only for the subset of variant-gene pairs and does not assess their significance. In the second step, SURGE treats the latent contexts as observed variables and performs standard interaction eQTL calling across all testable variant-gene pairs using either a standard linear model or linear mixed model.*

*In the first step, we use “representative variant-gene pairs” as the subset of variant-gene pairs to train SURGE on (in the original manuscript we referred to these variant-gene pairs as “independent variant gene-pairs but have changed the terminology to “representative variant-gene pairs” during the revision for maximal clarity). We want the representative variant-gene pairs to be a random sampling of all the context-specific eQTLs present in the data set. To achieve this, we encourage the representative variant-gene pairs to be independent of one another because we want SURGE to infer eQTL patterns that are persistent across the genome, not specific to a single gene or variant. For example, we do not want a SURGE context to represent a single LD block. We describe in detail our pipeline for selecting representative variant-gene pairs in the methods section entitled “Selection of representative variant-gene pairs used for optimization”. In summary, we select representative variant-gene pairs by selecting the top standard eQTLs found in the data set with the added constraint of limiting to a maximum of one variant-gene pair from each gene and one variant-gene pair from each variant.*

*In the second step, SURGE treats the latent contexts as observed and performs standard interaction eQTL calling (with respect to the SURGE latent contexts) for each testable variant-gene pair. This results in a p-value for each SURGE latent context across all variant-gene pairs. The methods section entitled “SURGE interaction-eQTLs” describes this in detail.*

*Next, we will summarize our multiple-testing correction procedure and describe new analyses included in the paper demonstrating the robustness of SURGE interaction eQTL detection to multiple testing correction assumptions. We assessed multiple-testing significance of SURGE interaction eQTLs using the empirical False Discovery Rate (eFDR). Briefly, eFDR establishes significance by comparing the observed SURGE interaction eQTL p-values with an empirical null distribution of p-values. We generated the empirical null distribution by permuting the genotype of each individual in the eQTL data set and re-running the entire SURGE interaction eQTL calling pipeline (including inference of SURGE latent contexts) using permuted genotype data. eFDR analysis was performed independently for each SURGE latent context. We view this choice analogous to the decision made in GTEx Consortium et al. Science 2020 to perform standard eQTL analysis multiple testing correction in each tissue independently and the decision made in Kim-Hellmuth et al. Science 2020 to perform interaction eQTL analysis multiple testing correction in each (tissue, cell-type) pair independently. The full details to our approach to multiple-testing correction are described in the following excerpt from the methods section entitled “Calibration of SURGE interaction eQTLs using permutation analysis”, which we have revised for clarity:*

*“P-values resulting from SURGE interaction-eQTL analysis are potentially inflated due to SURGE interaction eQTLs being identified from the same data used to learn the SURGE latent contexts. This statistical phenomenon is known as “double-dipping” and there exist well-studied approaches to ensure statistical calibration in the presence of “double-dipping” [41–44]. We used the empirical False Discovery Rate (eFDR) [45] to generate well-calibrated SURGE interaction eQTLs. eFDR establishes significance by comparing the observed SURGE interaction eQTL p-values with an empirical null distribution of p-values.*

*In more detail, we use a conservative, permutation analysis to generate an empirical null distribution of gene-level p-values that can be used to calibrate the observed gene-level p-values. The permutation analysis consisted of (1) permuting genotype of each individual, (2) re-optimizing SURGE latent contexts (U) using the permuted genotype data, and (3) calling SURGE interaction-eQTLs with the permuted genotype data and the SURGE latent contexts learned using the permuted genotype data.*

*To permute genotype, we generated a single permutation of genotype that was used across all analyzed variants to ensure we did not break the correlation structure across variant-gene pairs. In addition, we only permuted genotype across individuals, not RNA samples, to ensure we preserved sample repeat structure. This means that multiple RNA samples from the same individual will always have the same genotype values in the permutation run. Lastly, similar to previous permutation experiments on linear-interaction effects [8,46], we only permuted the genotype variable in the interaction term while leaving the fixed effect of genotype un-permuted (for both SURGE optimization and SURGE interaction-eQTL calling).*

*Given both the observed and permuted SURGE interaction eQTL p-values, we generated gene-level p-values using Bonferonni correction for both observed and permuted interaction eQTLs. We then evaluated genome-wide significance of the observed gene-level p-values using empirical FDR (eFDR) [45] calibrated with the permuted gene-level p-values. This approach was performed independently for each SURGE latent context. We will refer to this approach as the “per-context eFDR”.”*

*In response to the reviewer’s question, and for completeness, we now include an alternative multiple testing procedure where the eFDR is computed jointly across all tested SURGE interaction eQTLs in all SURGE latent contexts. We added the following text to the methods section entitled “Calibration of SURGE interaction eQTLs using permutation analysis”:*

*“Specifically, we evaluated genome-wide significance of the observed (gene, SURGE context) pair pvalues using eFDR calibrated using the permuted (gene, SURGE permuted context) pair pvalues. We will refer to this approach as the “all-context eFDR”. However, in concordance with previous multiple testing-correction procedures for multi-context eQTL calling [5,24], we recommend using the per-context eFDR.”*

*We now include supplementary tables containing the number of genes with a significant SURGE interaction eQTL for both the per context eFDR approach (Table S1 and Table S6) and the all context eFDR approach (Table S2 and Table S7). Relative to the all-context eFDR approach, the per-context eFDR approach identifies more genes with a significant SURGE interaction eQTL in the contexts with the most signal and fewer genes in the contexts with the least signal. Importantly, both approaches result in hundreds of identified eGenes.*

3. Is the SURGE model fitted with the fixed effects included?

*Yes. SURGE jointly infers fixed effects from known covariates, fixed effects from genotype, random effects from sample repeat structure, the SURGE latent contexts, and the interaction eQTL effect sizes of the SURGE latent contexts. Details regarding inference are described in the methods sections entitled “SURGE model overview” and “SURGE optimization”. We have revised a sentence in the second paragraph of the Results to clarify this point. The sentence now reads:*

*“To more accurately infer SURGE latent contexts and increase power to detect context-specific eQTLs, SURGE jointly controls for the effects of known covariates as well as sample repeat structure induced by assaying multiple measurements (such as many cells) from the same individual on gene expression when inferring latent contexts (see Methods).”*

4. How did the authors arrive at a PVE > 1e-5 cut off?

*We have updated supplementary figures S4 and S15 to motivate our choice of 1e-5 and to demonstrate the limited reliance of our results to this exact threshold. Specifically, these figures now show the proportion of expression variance explained (PVE) for all latent contexts optimized by SURGE, not just for the latent contexts that pass the filtering step (shown previously). The removed contexts explain a very small proportion of the expression variance and explain comparable levels to those obtained from permuted data. The exact value of 1e-5 was chosen empirically as it separated the clearly null contexts from those with signal in both eQTL data sets, and behaved well in simulations (Fig. 1D).*

*For additional interpretability, these figures (S4 and S15) also now show the “Proportion of SURGE-mediated variation explained” (PSMVE; see Methods) by all latent contexts. The SURGE-mediated expression variance is the variance in gene expression explained by any of the SURGE latent contexts. As can be observed in Fig. S4 and S15, the removed latent contexts explained a very small proportion of the total SURGE-mediated expression variance (this result can be attributed to the context-specific ARD prior distributions; see Methods). Specifically, the removed SURGE latent contexts explained in total only .08% and .2% of the total SURGE-mediated expression variance for the 10-tissue GTEx analysis and single cell PBMC analysis, respectively.*

5. What is the run time of this model, also in the complete mode (i.e. not the fitting step)?

*We have created a new supplementary figure (Fig. S1) that summarizes SURGE’s empirical run time for both SURGE optimization and for SURGE interaction eQTL calling. SURGE inference takes approximately 1.5 hours on a single core using 5000 eQTL samples and 19 hours on a single core using 20000 eQTL samples. Both tasks (SURGE optimization and interaction eQTL calling) could be parallelized in the future to decrease runtime. We note this as a limitation in the discussion:*

*“Fifth, SURGE cannot be reasonably scaled to more than 50,000 expression samples (Fig. S1). This does not present a concern for existing eQTL data sets, however, SURGE optimization could be extended in the future, if needed, to scale to arbitrarily large eQTL data sets using parallel programming or stochastic optimization [37]”*

6. Why were 80 factors corrected out of the expression data in GTEx wouldn't this remove a lot of contexts?

*We controlled for the effects of 80 gene expression principal components on expression in GTEx. This is distinct from controlling for the effects of the interaction between genotype and the 80 gene expression principal components on expression, which would remove a lot of discoverable contexts. As SURGE infers contexts whose interaction with genotype results in an effect on gene expression, it is possible for SURGE to infer a latent context that is highly related to one of the (controlled-for) expression principal components. Indeed, we show that SURGE latent contexts are often well explained by expression principal components that have been controlled for (Fig. S11 and Fig. S22).*

*The choice to control for 80 gene expression principal components was motivated by the recent GTEx eQTL paper (GTEx Consortium et al. Science 2020). They recommended controlling for 60 PEER factors when analyzing the largest sample size GTEx tissues (and fewer PEER factors for the smaller sample size tissues). As our primary GTEx analysis consisted of an aggregation of RNA-seq samples across 10 tissues and resulted in a combined sample size larger than any individual GTEx tissue, we opted to increase the number expression PCs to 80. Recent work (Zhou et al. Genome Biology 2022) motivated our choice to use expression principal components instead of PEER factors.*

7. For the GTEx analysis did the authors also include a random effect term for the eQTL mapping in GTEx? in the SC analysis it is specifically mentioned but not for the other analysis.

*We did not control for a random effect term related to sample repeat structure when applying SURGE to the 10-tissue GTEx data. While this analysis contained multiple RNA-seq samples from the same individual (due to GTEx extracting multiple tissue samples from the same individual), there were only 5.1 (on average) samples per individual. We initially tried running lme4 (the gold standard R linear mixed model package) to detect standard eQTLs in GTEx, but the model failed to converge for a large fraction of tested variant-gene pairs. We believe these models failed to converge because there were too few repeated measurements to estimate the variance of the random effect intercept.*

*For complete transparency, we also ran SURGE with a random effect intercept term for sample repeat structure on the 10-tissue GTEx data. We have added a new supplementary figure (Fig. S7) that shows the high levels of correlation between the identified SURGE latent contexts between the two models. More than 75% of the variance (adjusted R-squared) in 8 out of the 15 contexts identified in the main analysis (no random effect intercept) can be explained by the 11 SURGE latent identified in the secondary analysis (with a random effect intercept).*

*We have updated the methods section entitled “Application of SURGE to GTEx samples from 10 tissues: SURGE optimization” to reflect this information.*

**2nd round**

**Reviewer 1**

The revised manuscript by Strober and colleagues addresses the main points raised by this reviewer.

**Reviewer 2**

Thanks for the extensive reply to the questions and comments, and sorry for the long wait. I'm mostly very happy with the replies and the current state of the manuscript.

I do believe that the comparison with the current state of the art is too limited. I might have missed the link but I miss the explicit comparison to Nathan and Cuomo? (or even the PCA based methods) I see the comparison in the colocalization part, but where is the direct comparison?

Also strictly speaking, these are not PCA based methods; CellRegMap is used mostly on MOFA factors, and Nathan et al describe CCA variables. I’m wondering if these factors more closely relate to the SURGE factors then PCs. Also in the updated text in the first paragraph, it states only PCA, nothing about MOFA / CCA factors.

Lastly a minor point:
I now had the chance to look into the Vochteloo method and strictly spoken the comment about Vochteloo et al is not correct. Their method only updates the factors based on the interaction term (not on the fixed effect term (or both) which is described in the text).

**Authors’ response**

1. I do believe that the comparison with the current state of the art is too limited. I might have missed the link but I miss the explicit comparison to Nathan and Cuomo? (or even the PCA based methods) I see the comparison in the colocalization part, but where is the direct comparison?

*We agree it is important to compare SURGE to existing interaction eQTL calling approaches. We have extensively compared SURGE interaction eQTLs with expression PC interaction eQTLs. Specifically, we report the pairwise correlations between SURGE latent contexts and expression PCs (Fig S11 and Fig S22) and demonstrate that SURGE identifies slightly more interaction eQTLs than expression PCs (Fig S6 and Fig S17). Most importantly, the interaction eQTLs identified by SURGE are not identical to the interaction eQTLs identified with expression PCs, and we show that SURGE interaction eQTLs identify significantly more GWAS trait colocalizations than expression PC interaction eQTLs (Fig 3C), with most of the novel colocalizations discovered in SURGE latent contexts not captured by expression PCs (Fig S22 and Fig S25).*

*We have updated the Methods section entitled “Expression PC interaction-eQTLs” to include justification for the comparison with expression PC interaction eQTLs instead of the interaction eQTL approaches used in Nathan et al. or Cuomo et al. Please see the response to comment 2 below for more complete explanation for why these methods were not compared.*

2. Also strictly speaking, these are not PCA based methods; CellRegMap is used mostly on MOFA factors, and Nathan et al describe CCA variables. I'm wondering if these factors more closely relate to the SURGE factors then PCs. Also in the updated text in the first paragraph, it states only PCA, nothing about MOFA / CCA factors.

*Thank you for the opportunity to explain this point.*

*The CCA latent factors used in Nathan et al. can only be generated from multimodal data sets consisting of paired gene expression and protein measurements. SURGE was developed for application on traditional single-cell eQTL data sets, and a comparison to CCA latent factors, which would require multimodal data, is beyond the scope of this paper.*

*Regarding CellRegMap, we have added the following paragraph do the Methods section entitled “Expression PC interaction-eQTLs” motivating our comparison to expression PC interaction eQTLs, and clarifying the high levels of concordance between expression PCs and MOFA factors:*

*“Expression PC interaction eQTLs are closely related to CellRegMap interaction eQTLs [21], except CellRegMap uses MOFA factors [25] instead of gene expression PCs and CellRegMap treats the interaction eQTLs effect sizes as random effects instead of fixed effects. It was shown in Appendix Figure S5 of the CellRegMap manuscript [21] that MOFA factors were highly correlated with gene expression PCs and CellRegMap interaction eQTL p-values were highly concordant regardless of whether gene expression PCs or MOFA factors were used. Ultimately, we used our own implementation of expression PC interaction eQTL calling simply to minimize modelling assumption differences with SURGE and allow a focused comparison between the use of SURGE latent contexts and expression PCs in interaction eQTL calling.”*

*In addition, we have modified the first paragraph of the results section to emphasize that existing interaction eQTL methods were not limited to expression PCs:*

*“Relatedly, others have tested the interaction between genotype and reduced dimension expression features (such as gene expression principal components or MOFA factors [25]) on gene expression [13,21], but this imposes the limiting assumption that all contexts that modulate the genetic regulation of gene expression can be explained by reduced-dimension features of gene expression and limits eQTL discovery to contexts with large effects on broad gene expression patterns.”*

3. Lastly a minor point: I now had the chance to look into the Vochteloo method and strictly spoken the comment about Vochteloo et al is not correct. Their method only updates the factors based on the interaction term (not on the fixed effect term (or both) which is described in the text).

We thank the reviewer for noticing this mistake. We have updated the main text to accurately describe the method developed in Vochteloo et al., while still highlighting differences between SURGE and existing methods:

“SURGE is unique from these methods in that it identifies contexts whose linear interaction with genotype explain the most variation in single-cell gene expression levels. Vochteloo et al. [27] identifies contexts using an iterative algorithm such that the interaction between the context and genotype maximize expression variation in only the previous iteration’s genome-wide significant context-interaction eQTLs. Contexts identified by this approach may not directly correspond to contexts whose interaction with genotype maximally explains variation in gene expression. Furthermore, this approach does not model sample-repeat structure and was not developed for application on single-cell eQTL data.”
